# A SNP panel for identification of DNA and RNA specimens

**DOI:** 10.1186/s12864-018-4482-7

**Published:** 2018-01-25

**Authors:** Soheil Yousefi, Tooba Abbassi-Daloii, Thirsa Kraaijenbrink, Martijn Vermaat, Hailiang Mei, Peter van ‘t Hof, Maarten van Iterson, Daria V. Zhernakova, Annique Claringbould, Lude Franke, Leen M. ‘t Hart, Roderick C. Slieker, Amber van der Heijden, Peter de Knijff, BIOS consortium, R. Jansen, J. B. van Meurs, B. T. Heijmans, D. I. Boomsma, J. van Dongen, J. J. Hottenga, P. E. Slagboom, H. E. D. Suchiman, M. van Iterson, E. W. van Zwet, P. A. ‛t Hoen, R. Pool, M. M. van Greevenbroek, C. D. Stehouwer, C. J. van der Kallen, C. G. Schalkwijk, C. Wijmenga, A. Zhernakova, E. F. Tigchelaar, M. Beekman, J. Deelen, D. van Heemst, J. H. Veldink, L. H. van den Berg, C. M. van Duijn, B. A. Hofman, A. G. Uitterlinden, P. M. Jhamai, M. Verbiest, M. Verkerk, R. van der Breggen, J. van Rooij, N. Lakenberg, H. Mei, J. Bot, D. V. Zhernakova, P. Van’t Hof, P. Deelen, I. Nooren, M. Moed, M. Vermaat, M. J. Bonder, F. van Dijk, M. van Galen, W. Arindrarto, S. M. Kielbasa, M. A. Swertz, A. Isaacs, L. Franke, Peter A. C. ’t Hoen

**Affiliations:** 1 Department of Human Genetics, Leiden University Medical Center, Postzone S4-P, PO Box 9600, 2300 RC Leiden, The Netherlands; 2 Sequencing Analysis Support Core, Leiden University Medical Center, Leiden, The Netherlands; 3 Molecular Epidemiology Section, Leiden University Medical Center, Leiden, The Netherlands; 4 Department of Genetics, University Medical Centre Groningen, Groningen, The Netherlands; 5 Department of Molecular Cell Biology, Leiden University Medical Center, Leiden, The Netherlands; 6 Amsterdam Public Health Research Institute, VU University Medical Center, Amsterdam, The Netherlands; 7 Department of Epidemiology and Biostatistics, VU Medical Center, Amsterdam, The Netherlands; 8 Department of General Practice and Elderly Care Medicine, VU Medical Center, Amsterdam, The Netherlands; 9 Centre for Molecular and Biomolecular Informatics, Radboud Institute for Molecular Life Sciences, Radboud University Medical Center, Nijmegen, The Netherlands

**Keywords:** Genetic variation, Sample tracking, Mix up samples, Biobanking, Forensics

## Abstract

**Background:**

SNP panels that uniquely identify an individual are useful for genetic and forensic research. Previously recommended SNP panels are based on DNA profiles and mostly contain intragenic SNPs. With the increasing interest in RNA expression profiles, we aimed for establishing a SNP panel for both DNA and RNA-based genotyping.

**Results:**

To determine a small set of SNPs with maximally discriminative power, genotype calls were obtained from DNA and blood-derived RNA sequencing data belonging to healthy, geographically dispersed, Dutch individuals. SNPs were selected based on different criteria like genotype call rate, minor allele frequency, Hardy–Weinberg equilibrium and linkage disequilibrium. A panel of 50 SNPs was sufficient to identify an individual uniquely: the probability of identity was 6.9 × 10^− 20^ when assuming no family relations and 1.2 × 10^− 10^ when accounting for the presence of full sibs. The ability of the SNP panel to uniquely identify individuals on DNA and RNA level was validated in an independent population dataset. The panel is applicable to individuals from European descent, with slightly lower power in non-Europeans. Whereas most of the genes containing the 50 SNPs are expressed in various tissues, our SNP panel needs optimization for other tissues than blood.

**Conclusions:**

This first DNA/RNA SNP panel will be useful to identify sample mix-ups in biomedical research and for assigning DNA and RNA stains in crime scenes to unique individuals.

**Electronic supplementary material:**

The online version of this article (10.1186/s12864-018-4482-7) contains supplementary material, which is available to authorized users.

## Background

Over the past years, DNA profiles have found increasing use in the identification of human samples: they are ideal for sample tracking in biomedical studies and forensic investigations. In recent years, joint analysis of DNA and RNA has proven to be valuable: 1) Forensic investigations where RNA profiles may complement DNA profiles, and may be used to establish the tissue origin of the specimen [1], wound age, post-mortem interval, and the age of stains [2–5]; 2) Population research in which the genetic component of gene expression is studied.

Single nucleotide polymorphisms (SNPs) and other genetic markers like mitochondrial haplotypes, Y chromosomal markers and short tandem repeats (STRs) are all used for individual identification. Mitochondrial DNA (mtDNA) is found in both females and males but it is inherited only through the mother, which makes it impossible to differentiate between mothers and offspring [1, 6]. Y chromosome, a male-specific part is widely used in genetics studies and forensic data analysis particularly in cases of sexual assault, although identification of individuals using Y chromosome DNA analysis is limited to non-related subjects [6–8]. STRs, regions with short repeat units (usually 2–6 base pairs in length), are highly informative because of the large number of alleles present even in genetically rather homogeneous populations [9–11]. Despite their high discriminatory power, they have some limitations such as required large amplicon sizes, high mutation rates, and the presence of artefacts, which can negatively influence the downstream analysis [12, 13]. To overcome these limitations, SNPs have been more recently introduced for individual identification [14].

SNPs are defined here as single nucleotide substitutions that occur in more than 1 % of the general population. SNP assays can be used for multiple types of genetics studies. A recent review by Kayser and de Knijff provides an overview of recent advances in the use of SNPs for forensic investigation [15]. Many studies have discussed the advantages of SNPs compared to STRs, including low mutation rates, fast genotyping, high abundance in the genome, and straightforward detection using high-throughput technologies [16–20]. Kidd et al. [21] described a set of SNPs with high heterozygosity and low frequency variation in different populations, both helpful characteristics for an individual identification panel. In the last decade, several research groups have found valuable alternative individual identification SNP (IISNP) panels for different populations in the world [14, 22–26]. Pakstis et al. [20] selected 86 unlinked candidate IISNPs for 44 major populations across the world. Also, recently an IISNP panel for global forensic casework was established (Illumina, 2015). Moreover, research groups have tried to develop new SNP markers to improve their discrimination power using high throughput data sets [13, 27, 28]. However, these panels mostly consist of intragenic SNPs or SNPs in genes that are not expressed in blood or other tissues relevant for forensic identification. Therefore, there is no suitable panel that contains a minimum of informative SNPs that can be used on both DNA and RNA specimens.

To address the limitations of current IISNP panels, we present a small and powerful IISNP panel. When turned into a multiplex assay, this panel can be exploited for unequivocal identification of individuals in both DNA and RNA specimens in forensic investigations. Moreover the panel can be used to identify sample mix-ups in human gene expression studies, which have been demonstrated to severely affect the power of such studies [PMID: 21653519].

## Methods

### Sample collection and sequencing

Several Dutch biobanks contributed to sample collection of Dutch ancestry (with parents born in the Netherlands) within the Biobanking and Biomolecular Research Infrastructure-Netherlands (BBMRI-NL), established as a national node of the European BBMRI infrastructure in the Netherlands [29]. Our DNA datasets are derived from Genome of the Netherlands (GoNL), a whole-genome-sequencing effort within BBMRI-NL consisting of 250 representative parent-offspring families widely dispersed across the Netherlands (231 trios and 19 quartets, of which 11 had monozygotic twins and 8 had dizygotic twins), which aims to characterize DNA variation in the Dutch population. DNA-based genotype calls were derived from DNA isolated from blood [29].

The Biobank-based Integrative Omics Studies Consortium (BIOS Consortium) is also part of BBMRI-NL. Its aim is to create a large-scale data infrastructure and to bring together BBMRI researchers focusing on integrative omics studies in Dutch biobanks. The BIOS Consortium applies a functional genomics approach that integrates genome-wide genetic data with data on the epigenome and transcriptome. The RNA data were derived from individuals from seven Dutch biobanks participating in the BIOS Consortium (LL, LifeLines Cohort Study; LLS, Leiden Longevity Study; RS, Rotterdam Study; CODAM, Cohort on Diabetes and Atherosclerosis Maastricht; NTR, the Dutch Twin Registry; PAN, Prospective ALS study Netherlands). Globin RNAs were removed from whole blood and the polyA fraction of the remaining RNA was subjected to RNA sequencing using HiSeq2000 sequencers and were analyzed as described by [30]. RNA-Seq data are available in the European Genome-Phenome Archive (EGA) under accession: EGAD00001001623. Additionally, for each sample microarray-derived SNP data (Immunochip on all samples and at least one other GWAS array per sample) were generated by the biobanks [30–33]. Sequencing and the primary analysis of the data was performed within the BIOS and GoNL working groups. Variant calling was done using Samtools (v1.1) and Varscan (v2.3.7) and genotype calling then was performed at the SNP sites. We have carried out further analysis based on RNA-based genotypes calling on 2115 samples (after removing related samples) (Additional file 1: Table S1).

### Filtering and identifying SNPs

**Fig. 1:**
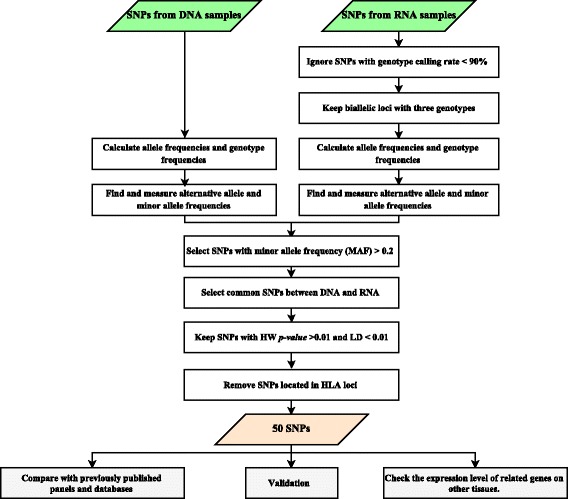
Flow chart of different steps to select panel of 50 SNPs and downstream analysis

Our strategy and criteria are shown in Fig. 1. Briefly, the allele frequencies for each SNP were calculated by genotype counting within population assuming each marker is a two-allele, co-dominant system. Genotype frequencies and maximum/minimum allele frequencies were then calculated based on allele frequencies. Reference alleles were extracted from Ensembl GRCh37 release 84 and alternative alleles were acquired for each SNP. Alternative allele frequencies (AAF) were measured and, minor allele frequency (MAF) was calculated based on the frequency of least common allele for each SNP in population. Then we have eliminated: 1) SNPs with genotype call rate less than 90%, 2) SNPs with more than three genotypes or more than two alleles, 3) SNPs with MAF less than 0.2 in both DNA-Seq and RNA-Seq data. To further filtration, the Hardy–Weinberg equilibrium (HWE), and linkage disequilibrium (LD) tests were used. Also, we have ignored SNPs located on human leukocyte antigen (HLA) loci where the SNP calls are prone to artefacts, particularly in NGS-derived datasets (Fig. 1). Finally, consistency was determined by analyzing the AAF in the RNA and DNA data from the same set of 2115 individuals (Additional file 1: Table S1), and 50 independent SNPs were selected with high MAF, high heterozygosity and no LD between them in our population.

The selected SNPs were compared with previously published panels such as SNPforID 52-plex, 75 Chinese SNPs, 30 Korean SNPs and 92 IISNPs [20, 23, 34, 35]. Furthermore, the AAF of the 50 SNPs were compared to 1000 Genomes, Exome Aggregation Consortium (ExAC), NHLBI GO Exome Sequencing Project (Go.ESP) and the 1000 Genomes phase-3 populations. Moreover, the predicted effect of our variants on protein sequence was extracted from Ensembl. R scripts implementing all these filtering steps.

### Statistical analysis

*Observed heterozygosity* and expected *heterozygosity* were estimated based on genotype counts and allele frequencies, respectively. So, deviation from HWE was determined by comparing the expected and observed number of individuals with each possible genotype using Fisher exact test. The *p-values* of the HW tests were corrected for multiple testing using the method developed by Benjamin and Hochberg [36], implementing a false discovery rate (FDR) of 1%.

To evaluate statistical independence of the SNPs, r^2^ was calculated for all unique pairwise combinations of the common SNPs between DNA-Seq and RNA-Seq data. The LD values were used to determine whether there was any evidence for significant linkage among the markers. In addition, heterozygosity, fixation index (also called the inbreeding coefficient, is defined as (He – Ho) / He (where He is expected heterozygosity and Ho is observed heterozygosity). It may range from − 1 to + 1. Values close to zero are expected under random mating, while substantial positive values indicate inbreeding or undetected null alleles. Negative values indicate excess of heterozygosity, due to negative assortative mating, or selection for heterozygotes.), polymorphism, Hardy–Weinberg equilibrium and probability of identity (provides an estimate of the average probability that two unrelated individuals, drawn from the same randomly mating population, will by chance have the same multilocus genotype. It is also called Population Match Probability), were checked using the Excel add-in GenAlEx [37, 38].

### Validation

The performance of the 50 SNP panel was examined by two strategies. First, the ability of the 50 SNP panel to identify sample mix-ups was checked in the second batch of samples from the BIOS project with 1357 independent samples, not used for the panel identification, using the same genotype calling methods as described in the first phase of the project. In the second strategy, the SNP panel was compared with a much larger set of 2622 SNPs. The 2622 SNP panel was selected as follows: 1) All ExAC v0.3 biallelic SNPs were selected [39]; 2) overlapped with exonic regions from Ensembl v75 and 1000 Genomes phase 1 high confidence SNPs, and 3) filtered on MAF > 0.4. Simultaneously, 1) SNPs that were called using Unified Genotyper in the LL subset of GoNL RNASeq samples were selected, and 2) filtered on a Unified Genotyper quality score of > 100,000. The final set of 2622 SNPs was constructed by overlapping the ExAC based and RNA-Seq based SNP lists. Both 2622 SNP and 50 SNP panels were used to evaluate the ability for sample and mismatch identification in samples from the Diabetes Care System (DCS) cohort [40], where there were 562 RNA-Seq as well as 3428 GWAS samples imputed using the HapMap reference panel. Genotype calling at all genomics coordinates was performed for the two SNP panels in both RNA-Seq and imputed GWAS samples. Briefly, our RNA-Seq pipeline (http://biopet-docs.readthedocs.io/en/latest/pipelines/gentrap/) was used to obtain genotype calls for 562 RNA-Seq samples. Further, for imputed GWAS samples, genotyping using the Human Core Exome chip was performed according the manufacturers protocol (Illumina Inc. San Diego, Ca, USA). Then, quality control was demonstrated using following settings: a cut-off of 99% for genotyping call rate, gentrain and cluster score < 0.6 and 0.4, respectively, and *p*-value cut-off for Hardy-Weinberg equilibrium was set at 10^− 4^. Consequently, imputation was done using SHAPEIT (v2.r644) and IMPUTE (v2.3.2). These two files were merged into one multi-sample VCF file that contained genotype information of total 3990 samples. All genomics coordinates specified by those two SNP lists were included in this VCF file. To compare these two SNP panels, we first calculated the pair-wise allele concordance scores for all 3990 by 3990 sample pairs by examining only the genomics coordinates specified in the 50 SNP panel. The allele concordance score (ranging from 0 to 1) for each sample pair was defined as the ratio between the number of identical alleles and the total number of alleles (100 alleles in total) through all 50 SNPs coordinates. In addition, the identification of the best matching GWAS samples for each RNA-Seq sample was examined with a minimal allele concordance score of 0.8. Multiple best GWAS sample hits for one RNA-Seq sample was possible as there have been repeated GWAS measurements performed on the same person. Then we performed the same steps using the long 2622 SNP panel to identify the best matching GWAS samples for each RNA-Seq sample.

## Results

### Data and SNP identification

In previous studies, most of the recommended individual identification SNP panels were generated based on DNA profiles and they mostly contain intragenic SNPs. Therefore, there is no efficient panel with informative SNPs which can be used for both DNA and RNA. To overcome these limitations, in this study both DNA and RNA-based genotype calls were used to find a small set of SNPs that can be used for identification of individuals. DNA-based genotype calls (19,562,004 SNP positions) and RNA-based genotype calls were made on 2115 individuals from four Dutch biobanks. Reliable RNA-based genotype calls were obtained for 507,975 SNP positions across four cohorts (Additional file 1: Table S1).

**Fig. 2:**
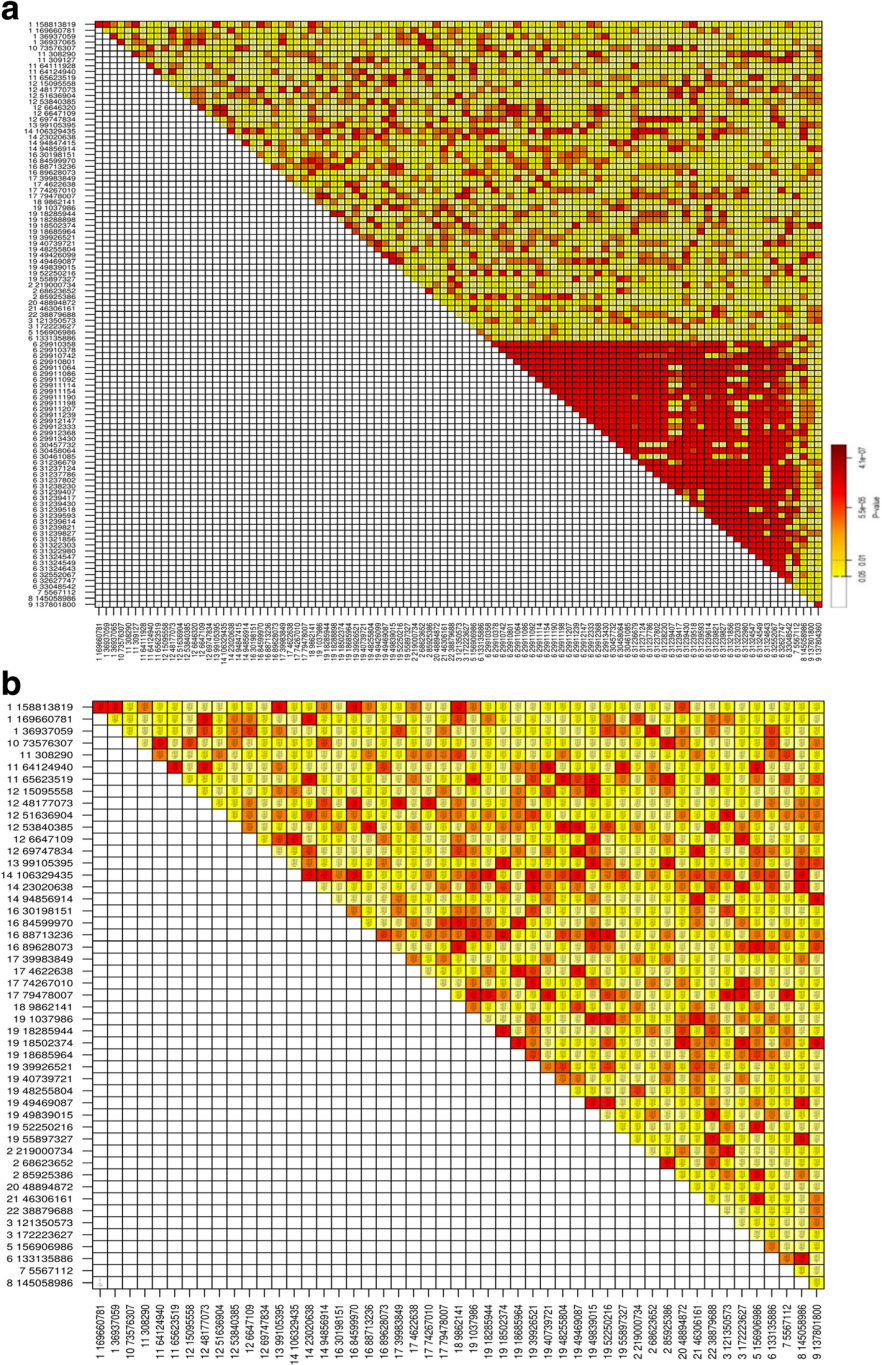
Pairwise LD comparisons of the set of **a** 100 SNPs before and **b** 50 SNPs after filtering for LD (r^2^ < 0.01). A color bar represents the *p*-values from the LD test

To find the most informative SNPs, we applied a number of filtering steps: details on the different filters applied and number of SNPs remaining can be found in the Method section, Fig. 1 and Table 1. After selection of SNPs with reliable genotype calls and high discriminative power (i.e. high MAF) and SNPs in Hardy–Weinberg equilibrium, 100 SNPs remained (Table 1). A final step to select the smallest informative set of SNPs for individual identification is absence of linkage disequilibrium between the SNP positions. To evaluate statistical independence of the SNPs, r^2^ was calculated for all unique pairwise combinations of 100 SNPs (Fig. 2a). SNP positions with r^2^ less than 0.01 were selected (Fig. 2b). We removed the least heterozygous SNP of any two SNPs, with LD and close genetic distance based on criteria of Kidd et al. [21] and Hwa et al. [23]. In addition, SNPs located in the HLA region were removed. Consequently, 50 SNPs were selected as a final panel to identify Dutch individuals (Table 1; Additional file 2: Table S2). The *r*^2^ values for the final list of 50 SNPs are very close to zero (median: 2.4 × 10^− 4^; average: 5.5 × 10^− 4^).

**Table 1:** Filtering steps with number of remaining SNPs

Filter steps	Number of SNPs	
	RNA	DNA
Total SNPs	507,975	19,562,004
Genotype calling rate > 90%	4876	–
Biallelic loci contain three genotypes	4672	–
MAF > 0.2	1263	3,077,712
Common SNPs between DNA and RNA	1023	
HW p-value > 0.01	100	
LD < 0.01 and Ignore SNPs located in HLA loci	50

Variant consequences for each position were extracted from Ensembl. As expected, most SNPs are located in exonic regions, because we selected SNPs which can be consistently detected in both RNA and DNA data. Twenty-four SNPs are located in the 3′-UTR, 22 SNPs in the coding region (14 synonymous, 8 missense), 3 SNPs in the 5′-UTR and 1 SNP in an intron (Additional file 2: Table S2).

### Characteristics and quality control of the 50 SNP set

**Fig. 3:**
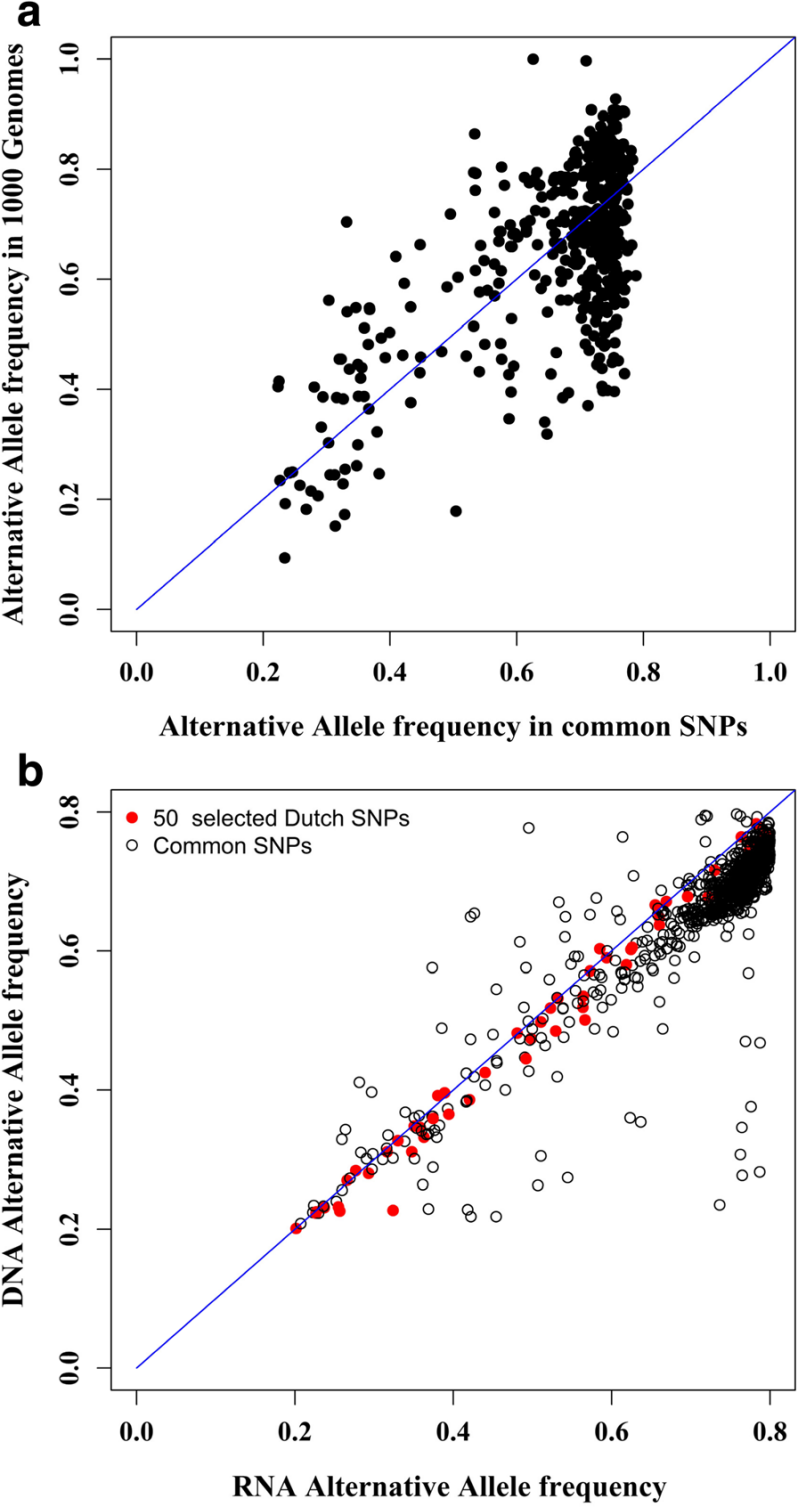
**a** Comparison of AAF between RNA (BIOS, x-axis) and DNA (GoNL, y-axis) data. **b** AAF comparison between Dutch population (common DNA/RNA, x-axis) and 1000 Genomes phase_3 populations (y-axis). Black points depict the common DNA/RNA SNPs before filtering and the red ones depict the 50 selected SNPs. *r* refers to Pearson correlation between data sets

The final selection of SNPs showed highly concordant AAF, for DNA and RNA-derived genotypes suggestive of the absence of bias in the genotype calls of these SNPs (Fig. 3a, r^2^ = 0.98). The correlation of MAF between RNA and DNA genotype calls was much higher for 50 selected SNPs (r^2^ = 0.81) than all confidently called SNP genotypes in both datasets (r^2^ = 0.6) (Additional file 3: Figure S1). The average MAF in the 50 selected SNPs was 0.35. The final selection of SNPs also had AAF > 0.15 in 1000 Genomes, but the AAF was generally higher in the Dutch population (Fig. 3b).

*Observed heterozygosity* and expected *heterozygosity* were measured based on genotype counts and allele frequencies, respectively. There was a high positive correlation (r^2^ = 0.96) with nearly equal frequency between expected heterozygosity and observed heterozygosity of 50 selected SNPs, suggesting that there was no large bias in detection of these SNPs. (Additional file 4: Figure S2 and Additional file 5: Figure S3).

Population genetic parameters were calculated to further characterize the SNPs in the panel (details in the Methods section) using the Excel add-in GenAlEx [37, 38]. The fixation indices were slightly negative for most SNPs (average: − 0.019), indicating some negative assortative mating with proper heterozygosity (Additional file 6: Figure S4 and Additional file 7: Figure S5).

### Discrimination power

**Fig. 4:**
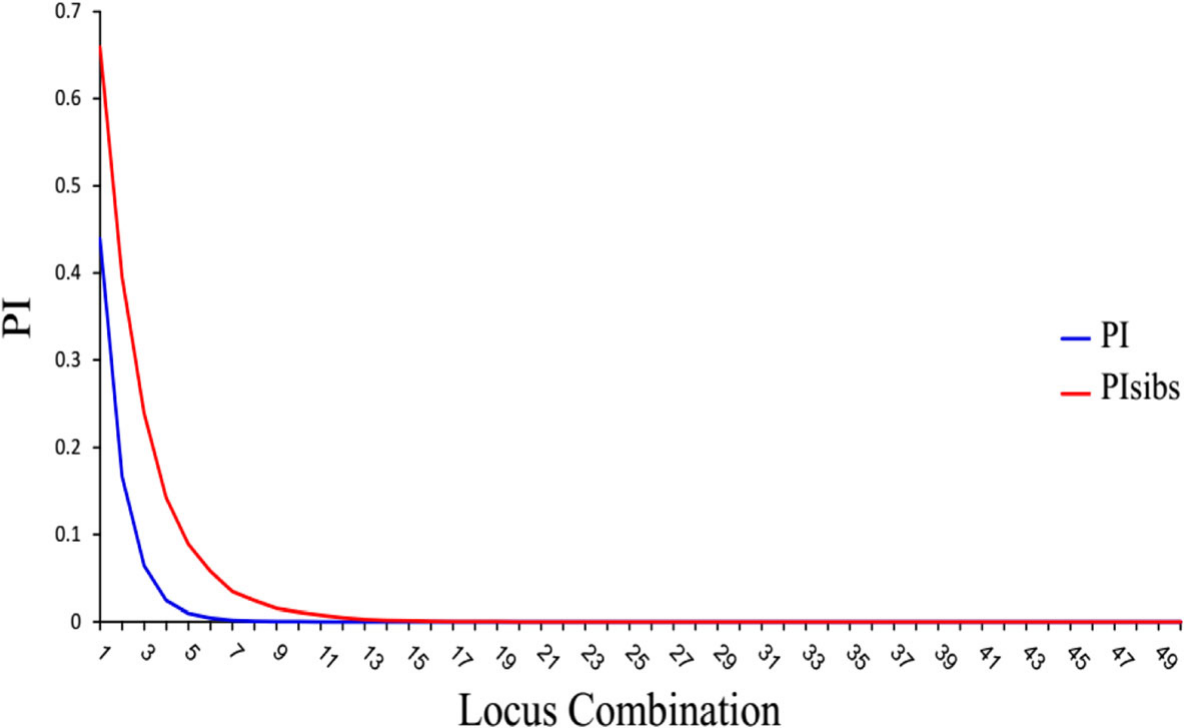
Probability of identity for 50 SNPs in 2115 samples**.** The blue line refers to PI between unrelated individuals. The red line refers to PI when related individuals are included in the samples (PISibs). The x-axis indicates the number of SNPs which are needed for identity when PI is zero

The probability of identity was analyzed for the panel of 50 SNPs in 2115 Dutch samples. The PI provides an estimate of the average probability of two independent samples having identical genotype calls. It is used to determine the minimum number of SNPs which are needed for identity calling. PI can be calculated under the assumption that all individuals are unrelated or under that the assumption that individuals may be related (PI-sibs). Figure 4 shows that at least 17 SNPs are required to achieve uniqueness in 2115 Dutch samples (PI is 3.3 × 10^− 7^ for unrelated individuals and PI-sibs is 4.4 × 10^− 4^). For our final list of 50 SNPs, the PI was 6.9 × 10^− 20^ and PI-sibs were 1.2 × 10^− 10^ (Fig. 4). This makes the marker set appropriate for tagging and tracking samples in large biomedical, association, and epidemiological studies.

### Population comparison

**Fig. 5:**
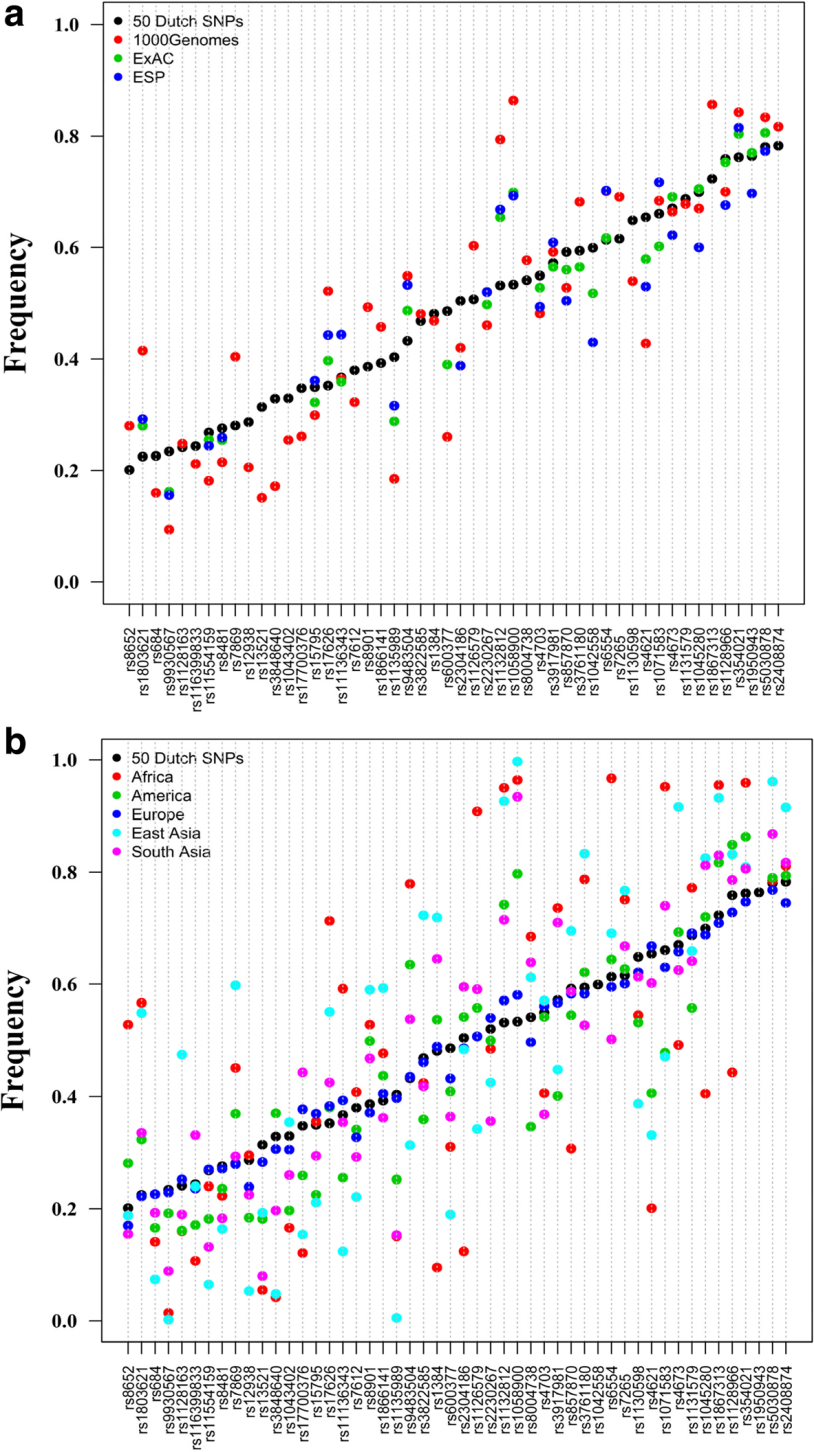
**a** AAF comparison of 50 selected SNPs in different populations. (Correlation between Dutch SNPs are: *r*^Dutch SNPs _ ExAC^: 0.94, *r*^Dutch SNPs _ ESP^: 0.87, *r*^Dutch SNPs _ 1000 Genomes^: 0.85. **b** Distribution of 50 selected SNPs in different populations. Correlation between Dutch SNPs is: *r* ^Dutch SNPs_Europe^: 0.99, *r*^Dutch SNPs_South Asia^: 0.87, *r*^Dutch SNPs_America^: 0.86, *r*^Dutch SNPs_East Asia^: 0.72, *r*^Dutch SNPs_Africa^: 0.58

To investigate the utility of our 50 SNP panel in other than the Dutch population, AAF of 50 SNPs were compared with the AAF in 1000 Genomes, ExAC and Go.ESP (Fig. 5a; Additional file 4: Figure S2). Figure 5a shows the AAF in Dutch SNPs are mostly consistent with those from ExAC. They are overall slightly higher in the Dutch population than in the populations catalogued in these three other databases, but consistently high for these databases which contain mostly individuals from European descent (Fig. 5a).

Even in non-European populations, such as the South Asian and American populations, most of the 50 SNPs had MAF > 0.2 (AAF between 0.2 and 0.8) (Fig. 5b; Additional file 2: Table S2). However, for the African population, our SNP panel was less effective, because 18 out of the 50 SNPs had MAF < 0.2 (Fig. 5b; Additional file 2: Table S2).

The selected SNPs were compared with previous published panels such as SNPforID 52-plex, 75 Chinese SNPs, 30 Korean SNPs and 92 IISNPs [20, 23, 34, 35]. There were no common SNPs between these panels and our 50 SNP panel.

### Validation of the 50 SNP panel

**Fig. 6:**
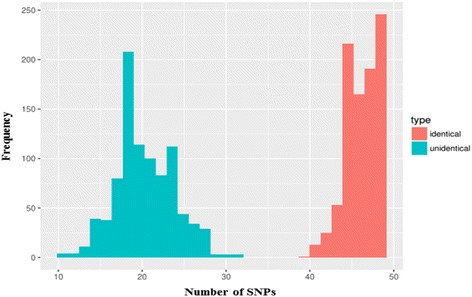
Distribution of the number of identical genotype calls in 1357 matching (red) and non-matching (random selection, blue) DNA and RNA samples

The ability of the SNP to uniquely identify individuals in both RNA and DNA level was evaluated by studying genotype concordance in an independent, paired set of 1357 DNA and blood-derived RNA samples. The distribution of concordant genotype calls for matched and unmatched DNA and RNA samples was clearly distinct and non-overlapping, with all matching samples having concordant genotype calls for at least 38 out of 50 of the SNPs (Fig. 6). Forty out of 50 SNPs demonstrated more than 90% concordant DNA and RNA genotype calls in this validation set of 1357 samples, whereas the minimum concordance observed was 65% for SNP rs2230267.

The ability of the 50 SNP panel to identify DNA and RNA sample concordance was compared with a bigger panel of 2622 SNPs in an independent study. The list of best GWAS hits for all 538 RNA-Seq samples with the RNA-Seq to GWAS mapping list provided by the study coordination center showed that the shorter 50 SNP panel detected more matching samples, while still reporting potential sample mix-ups (Table 2). Specifically, the number of samples where the genotype concordance test was indecisive was larger for the 2622 SNP panel.

**Table 2:** Number of sample matches in the DCS study using the 50 and 2622 SNP panels

Matching category(*)	50 SNP panel	2622 SNP panel
Passed_Matching	530	514
Failed_Matching	5	8
UnsureRNAseq	3	16
Total	538	538

“Passed_Matching”: contains RNAseq samples where the identified best GWAS hits are identical to the study’s mapping list

“Failed_Matching”: contains RNAseq samples where the identified best GWAS hits are different from the study’s mapping list

“UnsureRNAseq”: contains RNAseq samples for which no best GWAS hits were found based on our threshold (minimal allelic concordance score of 0.8)

## Discussion

We have established a pipeline to select a Dutch-specific SNP panel based on both DNA-Seq and RNA-Seq data. This panel consists of 50 SNPs with high heterozygosity, high MAF, low LD and robust detection in blood DNA and RNA (Table 1; Additional file 2: Table S2).

During the past years, various SNP panels have been published for individual identification [20–25, 41–47]. The SNPforID consortium developed 52 SNPs for individual identification [35]. Also, Kim et al. [34] developed a SNP-based individual assignment system containing 30 SNP loci for Korean individuals. Lou et al. [48] reported the performance of a 44 SNPs individual identification assay for Chinese. In addition, studies indicated considerable potential of high throughput platforms for SNP detection which could increase unprecedented discriminative power for human identification [49–51]. These panels are all based on DNA profiles and mostly contain intragenic SNPs which disqualify them for RNA-based genotype calling. Our set of SNPs is 98% exonic and can uniquely identify individuals in DNA and RNA profiles. Although use of coding SNPs in forensic DNA analysis may be restricted due to specific legislation in certain countries, this should not apply to highly polymorphic SNPs without any associations with appearance phenotypes.

There is no overlap between Dutch selected IISNPs and the established IISNP panels [13, 20, 23, 35]. One of our selected SNPs (rs1866141) is located in an intronic region of the highly expressed *GNLY* gene (MIM # 188855). Observing intronic SNPs among RNA-based calls analysis is common because of the presence of pre-mRNA, incomplete splicing or intron retention.

The 50 SNP panel showed better identification performance when it was compared with another panel containing 2622 SNPs (Table 2). Although the 50 SNP panel had superior performance in the Dutch and other populations of European descent, it is less optimal for individuals from African populations (Fig. 5b). There was no information for two SNPs (rs1950943 and rs1042558) in non-European populations.

RNA profiles can complement DNA profiles in research projects and forensic applications. In forensics, despite the presumed low stability of RNA, RNA profiles could not only identify individuals but also provide information about the type of tissues found at the crime scene, wound age determination, determination of the post-mortem interval and the functional status of cells as well as organs [2–5, 41, 52–59]. Unlike previous studies [20, 21, 25], 98% of our final SNPs located in exonic regions. For this reason, a SNP profiling assay for this 50 SNP panel can be an efficient method for individual identification in RNA and DNA stains from crime scenes. Analysis of RNA from crime scenes has demonstrated differences in RNA degradation rates. Although the SNPs from the 50 SNP panel are in high expressed genes and are robustly detected in biobank samples, their robust detection in forensic specimens containing partially to severely degraded RNA still needs to be demonstrated.

To address whether the SNP panel could also be used on RNA samples from tissues other than blood, the expression level of genes in which the SNPs are located was surveyed using GTEx portal [60]. While 25 genes were expressed ubiquitously and 17 genes were expressed in multiple other tissues, the expression of 8 genes (*MNDA* (MIM # 159553), *SELL* (MIM # 153240), *CSF3R* (MIM # 138971), *IFITM2* (MIM # 605578), *FPR1* (MIM # 136537), *CXCR2* (MIM # 146928), *GNLY* (MIM # 188855), *FCN1* (MIM # 601252)) was rather specific for whole blood, as their expression levels were near to zero in other tissues. Further, *IFI30* (MIM # 604664), contained the lowest gene expression in different tissues. As 17 SNPs are sufficient to identify an individual uniquely in the Dutch population it is assumed that our SNP panel could be effective for identification of individuals in RNA from different tissues than blood. However, when testing our panel on another SNP-chip genotype and RNA-Seq dataset, consisting of 36 samples from brain tissue, it appeared that many of the 50 SNPs had insufficient coverage for reliable RNA-based genotype calling in brain. This indicates that our 50 SNP panel needs optimization to be used for other tissues than blood and/or high sequencing depth.

Our SNP panel compared favorably to previously published panels in terms of discrimination power (probability of identity (PI)), even for closely related individuals. The FBI (USA) has selected 13 STR loci to serve as a panel for forensic investigations (CODIS, Combined DNA Index System). With this set of loci, the probability of a match between the profiles of two unrelated persons in a randomly mating population of Caucasian Americans PI is 2.97 × 10^− 15^ [61]. Also, the 52-plex SNP assay, which is now more routinely used in Europe, has a mean PI of 5.0 × 10^− 21^ for the European population [35]. The PI of our SNP panel was 6.9 × 10^− 20^ and 1.2 × 10^− 10^ in unrelated and related individuals, respectively, similar to the 52-plex SNP assay, far more discriminative than the 13 CODIS markers [61] and similar to the recently published study of 20 CODIS markers in Caucasian Americans [61–63] .

## Conclusions

We developed a first SNP panel based on both DNA and RNA data for the Dutch population. This panel contains 50 informative SNPs with high heterozygosity, low PI and close MAF and AAF frequencies in DNA and RNA. It will be useful for efficient sample identification/tagging in large biomedical, association, and epidemiologic studies, and for developing forensic profiling and kinship assays. Our panel will be useful for other European populations and can be considered in conjunction with other panels to develop a global IISNP panel with more markers.

## Additional files

Additional file 1: Table S1. Four biobanks (RNA-Seq) with number of different SNP positions and number of samples. (DOC 33 kb)

Additional file 2: Table S2. Characteristics of 50 selected SNPs. (CSV 15 kb)

Additional file 3: Figure S1. Distribution of MAF calculated from DNA and RNA data. (DOC 58 kb)

Additional file 4: Figure S1. Plot of the expected heterozygosity (x-axis) and observed heterozygosity (y-axis) for the 50 SNPs in the panel. Pearson correlation is 0.98. (DOC 54 kb)

Additional file 5: Figure S3. Individual heterozygosity across loci for each sample determined by GenAlEx software. (DOC 46 kb)

Additional file 6: Figure S4. The fixation index values of 50 selected SNPs. (DOC 54 kb)

Additional file 7: Figure S5. The average of population genetic parameters for 50 selected SNPs. (DOC 46 kb)

## Abbreviations

AAF: Alternative Allele Frequencies
BBMRI-NL: Biobanking and Biomolecular Research Infrastructure-Netherlands
BIOS Consortium: The Biobank-based Integrative Omics Studies Consortium
CODAM: Cohort on Diabetes and Atherosclerosis Maastricht
CODIS: Combined DNA Index System
DCS: Diabetes Care System
EGA: European Genome-Phenome Archive
ExAC: Exome Aggregation Consortium
FDR: False Discovery Rate
Go.ESP: NHLBI GO Exome Sequencing Project
GoNL: Genome of the Netherlands
HLA: Human Leukocyte Antigen
HWE: Hardy–Weinberg Equilibrium
IISNP: Individual Identification SNP
LD: Linkage Disequilibrium
LL: Life Lines Cohort Study
LLS: Leiden Longevity Study
MAF: Minor Allele Frequency
mRNAs: messenger RNAs
mtDNA: Mitochondrial DNA
NTR: the Dutch Twin Registry
PAN: Prospective ALS study Netherlands
PI: Probability of Identity
RS: Rotterdam Study
SNPs: Single Nucleotide Polymorphisms
STRs: Short Tandem Repeats
