# Supplementary material for: A SNP panel for identification of DNA and RNA specimens
Source: BMC Genomics. 2018 Jan 25;19:90. doi: 10.1186/s12864-018-4482-7 (PMC5785835; doi:10.1186/s12864-018-4482-7)
Supplement: Supplementary file 1 — Four biobanks (RNA-Seq) with number of different SNP positions and number of samples. (DOC 33 kb) [file 12864_2018_4482_MOESM1_ESM.doc]

| **Number of unrelated samples** | | **Samples** | **SNPs** | **Biobanks** |
| --- | --- | --- | --- | --- |
| 651 | | 658 | 1,080,591 | RS |
| 626 | | 630 | 1,044,703 | LL |
| 654 | | 720 | 1,140,784 | LS |
| 184 | | 191 | 624,880 | CODAM |
| 2,115 | **Total number of unrelated Samples** | | 507,975 | **Common SNPs between four biobanks** |

**Additional file 6: Table S1:** Four biobanks (RNA-Seq) with number of different SNP positions and number of samples
