# Supplementary material for: A SNP panel for identification of DNA and RNA specimens
Source: BMC Genomics. 2018 Jan 25;19:90. doi: 10.1186/s12864-018-4482-7 (PMC5785835; doi:10.1186/s12864-018-4482-7)
Supplement: Supplementary file 3 — Distribution of MAF calculated from DNA and RNA data. (DOC 58 kb) [file 12864_2018_4482_MOESM3_ESM.doc]

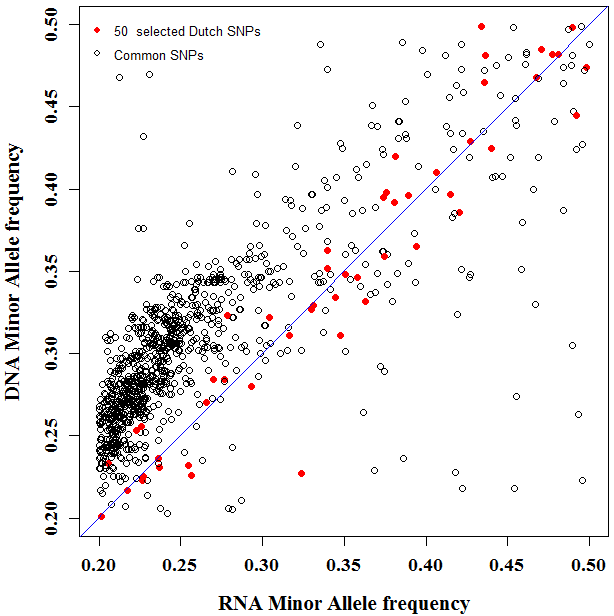


**Figure S1.** Distribution of minor allele frequencies calculated from DNA (y-axis) and RNA (x-axis) data. Black points depict 1,023 common SNPs and the red points depict 50 selected SNPs. The Pearson correlation between DNA and RNA MAF in 50 Dutch SNPs was 0.9 and in common SNPs was 0.78.
