# Supplementary material for: A SNP panel for identification of DNA and RNA specimens
Source: BMC Genomics. 2018 Jan 25;19:90. doi: 10.1186/s12864-018-4482-7 (PMC5785835; doi:10.1186/s12864-018-4482-7)
Supplement: Supplementary file 4 — Plot of the expected heterozygosity (x-axis) and observed heterozygosity (y-axis) for the 50 SNPs in the panel. Pearson correlation is 0.98. (DOC 54 kb) [file 12864_2018_4482_MOESM4_ESM.doc]

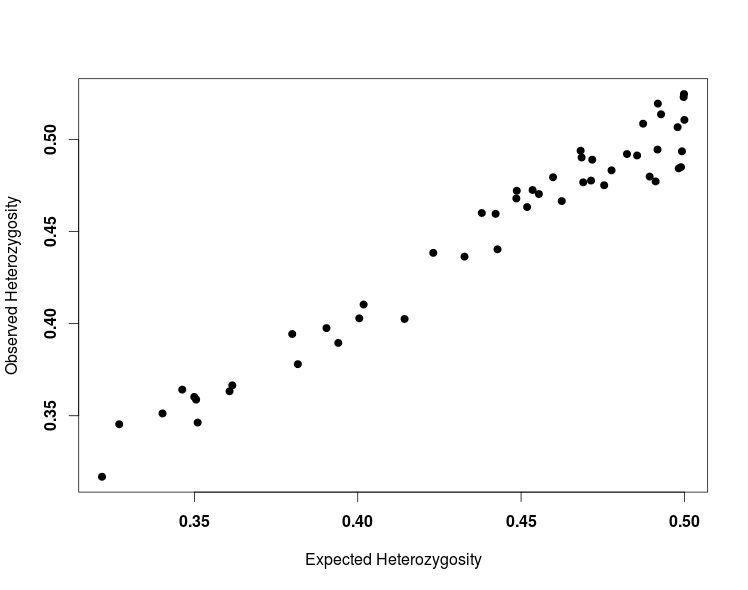
**Figure S2.** Plot of the expected heterozygosity (x-axis) and observed heterozygosity (y-axis) for the 50 SNPs in the panel. Pearson correlation is 0.98.
