# Supplementary material for: A SNP panel for identification of DNA and RNA specimens
Source: BMC Genomics. 2018 Jan 25;19:90. doi: 10.1186/s12864-018-4482-7 (PMC5785835; doi:10.1186/s12864-018-4482-7)
Supplement: Supplementary file 5 — Individual heterozygosity across loci for each sample determined by GenAlEx software. (DOC 46 kb) [file 12864_2018_4482_MOESM5_ESM.doc]

**Figure S3.**
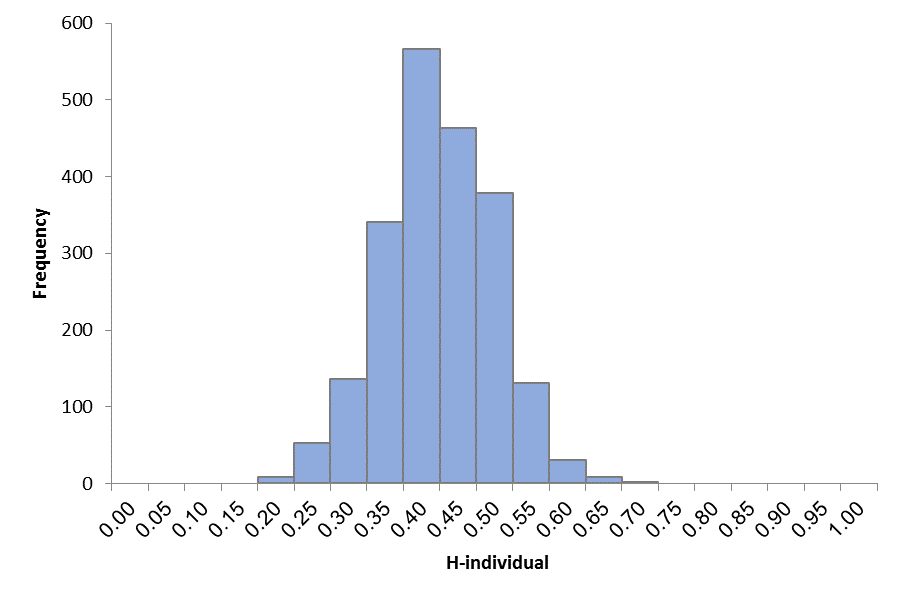
 Individual heterozygosity across loci for each sample determined by GenAlEx software. The distribution of heterozygosity loci across individuals is normal.
