# Supplementary material for: A SNP panel for identification of DNA and RNA specimens
Source: BMC Genomics. 2018 Jan 25;19:90. doi: 10.1186/s12864-018-4482-7 (PMC5785835; doi:10.1186/s12864-018-4482-7)
Supplement: Supplementary file 6 — The fixation index values of 50 selected SNPs. (DOC 54 kb) [file 12864_2018_4482_MOESM6_ESM.doc]

**
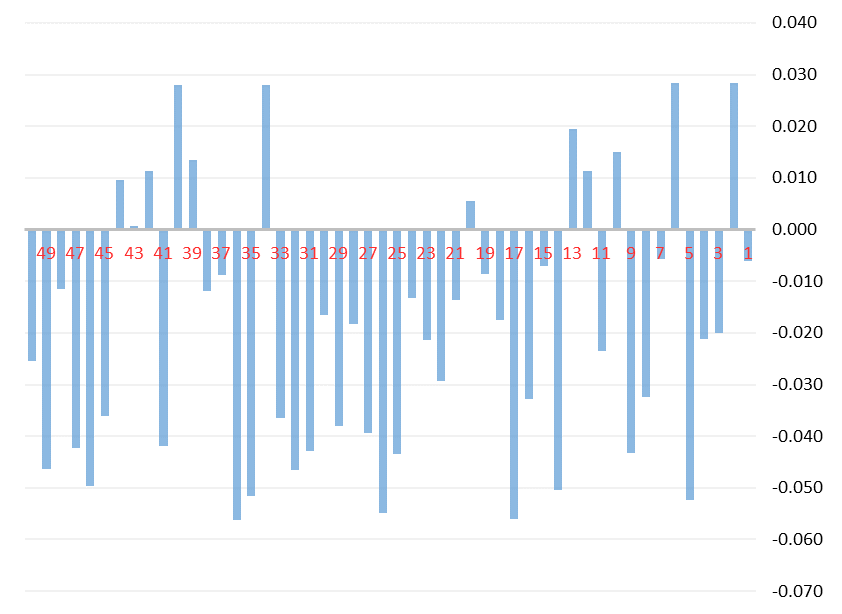
**

**Figure S4.** The fixation index values of 50 selected SNPs. Values close to zero are expected under random mating, while substantial positive values indicate inbreeding or undetected null alleles. Negative values indicate excess of heterozygosity, due to negative assortative mating, or selection for heterozygotes. Most loci showed negative values and the average was -0.019.
